# Model for End Stage Liver Disease (MELD) Score: A Tool for Prognosis and Prediction of Mortality in Patients With Decompensated Liver Cirrhosis

**DOI:** 10.7759/cureus.39267

**Published:** 2023-05-20

**Authors:** Isioma Emenena, Bright Emenena, Anthony G Kweki, Henry O Aiwuyo, John O Osarenkhoe, Ugoeze N Iloeje, Nosakhare Ilerhunmwuwa, Beatrice E Torere, Oluwasegun Akinti, Adegboyega Akere, Omuemu E Casimir

**Affiliations:** 1 Internal Medicine/Gastroenterology, Delta State University Teaching Hospital, Oghara, NGA; 2 Medicine, BU Clinic/Hospital, Warri, NGA; 3 Internal Medicine/Cardiology, Colchester Hospital, ESNEFT, Colchester, GBR; 4 Internal Medicine, Brookdale University Hospital Medical Center, Brooklyn, USA; 5 Medicine and Surgery, Igbinedion University Teaching Hospital, Benin City, NGA; 6 Internal Medicine/Cardiology, Federal Medical Centre, Yenagoa, NGA; 7 Internal Medicine, Brookdale Hospital Medical Center, Brooklyn, USA; 8 Internal Medicine, North Mississippi Medical Center, Tupelo, USA; 9 Medicine, College of Medicine, University of Ibadan, Ibadan, NGA; 10 Internal Medicine, University of Benin, Benin City, NGA; 11 Medicine, University of Benin Teaching Hospital, Benin City, NGA

**Keywords:** prediction of mortality, cirrhosis, decompensated liver disease, prognosis, meld score

## Abstract

Background: Decompensated liver disease has become a common occurrence in medical wards. It has become the third most common cause of death in medical wards. This high mortality rate has become a matter of concern. It is important that a reliable scoring system helps to stratify patients with liver cirrhosis who will require liver transplantation.

Objective: To determine the value of the Model for End-Stage Liver Disease (MELD) score in assessing the mortality of patients with decompensated liver cirrhosis over one month period (30 days).

Methods and materials: A longitudinal study was conducted. A total of 110 patients diagnosed with decompensated liver cirrhosis were recruited from the gastroenterology clinic and medical wards of the University of Benin Teaching Hospital (UBTH), Benin City. The patients were recruited consecutively and met the inclusion criteria for the study. Demographic data, history, clinical, biochemical, ultrasonographic, and liver biopsy findings were evaluated in the patients who participated in this study.

Results: The mean age of the patients was 57 ± 11.06 years. Out of the 110 study participants, a 2.9:1 male-to-female ratio was appreciated in the patient population, with a total of 82 males and 28 females.

Multiple logistic regression analysis identified MELD scores as an independent predictor of mortality in the studied patients. Predictive values of the MELD score for 1-month mortality which was analyzed using the receiver operating characteristic (ROC) curves showed that the MELD score had a sensitivity of 72.2% and positive predictive value of 93.6% with an area under the curve of 0.926 for all-cause mortality among decompensated liver cirrhosis patients.

Conclusion: MELD score is a good predictor of mortality among patients with decompensated liver cirrhosis over a 1-month (30 days) period.

## Introduction

Chronic liver disease (CLD) is defined as a disease of the liver resulting from an inflammatory, infiltrative, immunologic, circulatory, or metabolic injury to the liver which has been ongoing for 6 months or longer. It can also be defined as the destruction of the liver parenchyma leading to fibrosis and cirrhosis which has lasted more than 6 months. It is an asymptomatic progressive disease and is mostly fatal with five-year survival rates exhibiting a range from complete mortality to a maximum of 80% [1-3].

Chronic hepatitis B and C virus infections and chronic alcohol consumption are the most common etiologies of CLD; other causes include: non-alcoholic steatohepatitis (NASH), autoimmune hepatitis, primary biliary cirrhosis, primary sclerosing cholangitis, Wilson disease, hemochromatosis and alpha-1-antitrypsin deficiency; smoking, obesity, and diabetes mellitus are predisposing factors for CLD [4].

Chronic liver disease is the fifth leading cause of mortality globally and the third most common cause of death in the medical wards [5-6]. Approximately 29 million persons suffer from chronic liver conditions annually in European countries. In the United States, the prevalence of CLD was reported to be 63.9 cases/100,000 population. In Nigeria, a report from a hospital stated that liver diseases accounted for 7.9% of medical admissions with primary liver cancer and liver cirrhosis accounting for most of them [7-10].

Regardless of the etiology, the final pathway for CLD is liver cirrhosis which is a pathologic entity characterized by diffuse hepatic fibrosis with the replacement of normal liver architecture by regenerative nodules. The rate of progression varies in different patients depending on the etiology. It may take weeks in patients with complete biliary obstruction or decades in patients with chronic hepatitis C infection [11]. 

A cross-sectional study conducted by Apica et al. [12] noted that 17.6% had decompensated liver cirrhosis. This was similar to what was reported by Bell et al. [9] where a prevalence of 18% was noted. This was different from what was reported in the study by Patrick and Sha [11] where 41.3% was noted. Alcohol intake and viral hepatitis were the most common risk factors identified in these studies [9, 12-13].

The pathophysiology and pathology of CLD is complex. The progressive scarring of the tissue in the liver leads to altered hepatic flow and increased resistance to portal blood flow, leading to portal hypertension and loss of hepatic function. Patients with cirrhosis may be asymptomatic with physical findings of an enlarged/shrunken liver, enlarged/normal spleen, or both as in compensated state, or present with symptoms of hepatic dysfunction, portal hypertension, or both in a decompensated state [14].

The clinical manifestations of patients with CLD vary and depend on the etiology and stage at which the patient presents. It could be asymptomatic as seen in some cirrhotic patients [15], or features of decompensation could be seen; jaundice, abdominal and bilateral leg swelling, hepatomegaly and splenomegaly, and bleeding tendencies. These features are irrespective of the etiology. Signs and symptoms that point to the etiology can also be identified in the patient.

In a retrospective study conducted in the University of Nigeria Teaching Hospital, Ituku/Ozalla, Enugu, South-East Nigeria from 1995 to 2010, CLD was found to contribute to 7.0% of all mortalities in the medical wards. A similar study carried out in the University College Hospital, Ibadan, Nigeria, over 14 years showed that liver disease accounted for 12.1% of deaths and was the third most common cause of death in the medical wards [7, 16].

Cirrhosis, although strictly diagnosed histologically, a combination of clinical, laboratory, and imaging features can help confirm a diagnosis of liver cirrhosis. Symptoms like palmar erythema, Terry’s nails, digital clubbing, gynecomastia, spider telangiectasias, dilated abdominal vein, and parotid fullness are a pointer to liver cirrhosis. Patients with a history of CLD with gastroesophageal varices, ascites or hepatic encephalopathy are likely to have liver cirrhosis and liver biopsy may not be necessary to confirm the diagnosis of cirrhosis. An enlarged left hepatic lobe with splenomegaly in patients with peripheral stigmata of CLD as described, suggests cirrhosis, especially in the setting of thrombocytopenia and impaired hepatic synthetic function, e.g. hypoalbuminemia and prolongation of prothrombin time [11].

Serum aspartate transaminase (AST) and alanine transaminase (ALT) are two commonly ordered biochemical parameters which if elevated, may indicate significant liver disease. These parameters are part of the liver function test which is a frequently requested investigation in light of liver disease [17]. On ultrasonography, reduced liver span, nodularity, irregularity, and increased echogenicity are the hallmarks of a cirrhotic liver. In advanced disease, the gross liver appears small and multinodular, ascites may be detected, and Doppler flow can be significantly decreased in the portal circulation [18].

CT, MRI, liver biopsy, magnetic resonance angiography (MRA), shear wave elastographic (SWE) technique, and magnetic resonance elastography (MRE) also have a place in the evaluation of liver cirrhosis. A scoring system is used to best predict the clinical outcome of patients with cirrhosis. The MELD is one of the common scoring systems used [18]. It was developed in 2000 by Mayo Clinic and was adopted in 2002 as criteria for prioritizing patients on the liver transplant waiting list. It has, however, been modified in several ways to improve outcomes [19-20].

The MELD is based on three biochemical variables that are readily available, reproducible, and objective. These include: serum bilirubin, serum creatinine, and the international normalized ratio (INR) of prothrombin time. MELD is an excellent predictor of 3-month mortality among cirrhotic patients listed for orthotopic liver transplantation (OLT) [21]. Parameters in MELD scoring system are: MELD=3.78 x ln[serum bilirubin(mg/dL)] + 11.2 x ln[INR] + 9.57 x ln[serumCr(mg/dL)] + 6.43. The MELD score is reported as a whole number such that the above result is rounded off. If the patient has been dialyzed two times in the past 7 days then serum Cr = 4 mg/dL. Any value < 1 is given a value of 1.

Studies have shown that the MELD scoring system is most accurate in predicting short-term mortality among CLD patients [22-23]. In Nigeria currently, there are no centers that offer liver transplantation as a standard of care to decompensated patients. As such, these scoring systems have been kept abate. However, in the future when the need arises for a scoring system, a scoring system that has been researched will be highly regarded in prioritizing patients for liver transplantation.

## Materials and methods

A longitudinal study was carried out in the Gastroenterology Unit of the Department of Medicine, University of Benin Teaching Hospital (UBTH). 

An estimated study population of 110 participants was used in this study. Cochran’s formula was used for sample size determination. Participants for this study were patients with decompensated liver cirrhosis who presented at the Gastroenterology Clinic, admitted into the wards or at the Emergency Department. The patients satisfied the inclusion criteria and followed up for a month.

A total of 150 cirrhotic patients were screened for 8 months. Some 110 decompensated patients were recruited for the study. Cirrhotic patients who were not decompensated and those with co-existing morbidities like HIV/AIDS, heart failure, diabetes mellitus, renal disease, and hepatocellular carcinoma were excluded from this study. Patients were followed up for the 1-month duration of the study while on admission to the ward and those discharged home before the study duration elapsed were followed up on the phone.

A semi-structured interviewer-administered questionnaire was used to obtain socio-demographic data and information on medical history. A detailed evaluation was carried out on each patient to obtain optimal symptom history, medication history, family history, as well as general physical and abdominal examinations. Biochemical parameters of renal function (E/U/Cr), liver function tests, and clotting profiles were analyzed for the participants of this study.

The MELD score of the patients with decompensated cirrhosis was calculated using

MELD = 3.78 x ln [serum bilirubin (mg/dL)] + 11.2 x ln [INR] + 9.57 x ln [serumCr (mg/dL)] + 6.43

The MELD score was reported as a whole number such that the above result is rounded off. If the patient had been dialyzed two times in the past 7 days, then serum creatinine was taken as equal to 4 mg/dL (353.6 µmol/L). Any value < 1 was given a value of 1.

Ethical approval was obtained from the University of Benin Teaching Hospital Ethics and Research Committee with approval number ADM/E22/A/VOL.VII/14796.

Data were collated and analyzed using the Statistical Package for Social Sciences (SPSS) version 22.0 (IBM Corp., Armonk, NY). Categorical data such as socio-demographic characteristics for example age, sex, religion, etc were presented as frequencies and cross-tables. Continuous data such as PT and INR were presented as means and standard deviations. Results were presented as tables.

Multiple logistic regression was used to determine the predictors of 1-month mortality in patients with decompensated cirrhosis. Positive predictive value (PPV), negative predictive value (NPV), and sensitivity of the scoring systems were also calculated and compared using the following formulas.

NPV = specificity x (1-prevalence) / (specificity) x (1-prevalence) + (1-sensitivity) + prevalence

 and

PPV = specificity x prevalence/(specificity x prevalence) + (1-specificity) + (1-prevalence).

## Results

Demographic characteristics of the patients with decompensated liver cirrhosis

Out of the patients with decompensated liver cirrhosis, 92 (83.6%) survived, while 18 (16.4%) died during a period of 1-month follow-up. The mean age of the patients that died (62.67 ± 9.73 years) was significantly higher than those that survived (51.52 ± 12.39 years) (p=0.001). Males constituted 74.5%, while the females were 25.5% but gender did not determine mortality (p=0.349). Analysis of the level of education showed that number of deaths was significantly lowest among those patients with tertiary education (p=0.003) (see Table 1 below).

**Table 1:** Demographic characteristics of the patients. *t-test value SD, standard deviation

Characteristics				Total patients	Outcome of liver cirrhosis		ꭓ^2^	p-value
				(n=110) (%)	Survived (n=92)(%)	Died (n=18)(%)		
Overall mortality rate					92(83.6)	18(16.4)		
Mean age ± SD (years)				57.10±11.06	51.52±12.39	62.67±9.73	3.600*	0.001
Ages (years)							19.066	0.001
	<30			5 (4.5)	5 (5.4)	0(0.0)		
	30-39			11 (10.0)	11(11.9)	0(0.0)		
	40-49			26 (23.6)	26 (28.2)	0(0.0)		
	50-59			33 (30.0)	25(27.2)	8(44.4)		
	60-69			20 (18.2)	16 (17.4)	4(22.2)		
	70-79			13 (11.8)	9 (9.8)	4(22.2)		
	≥80			2 (1.8)	0 (0.0)	2(11.1)		
Gender							0.876	0.349
		Male		82 (74.5)	67(72.8)	15 (83.3)		
		Female		28 (25.5)	25(27.2)	3 (16)		
Marital Status							0.537	0.911
			Single	13 (11.8)	11(11.9)	2(11.1)		
			Married	87 (79.1)	72(78.3)	15(83.3)		
			Widowed	8 (7.3)	7(7.6)	1(5.6)		
			Separated	2 (1.8)	2(2.2)	0(0.0)		
Religion							2.138	0.144
			Christian	105(95.5)	89(96.7)	16(88.9)		
			Muslim	5(4.5)	3(3.3)	2(11.1)		
Occupation							2.542	0.770
			Professional	2(1.8)	2(2.2)	0(0.0)		
			Business	33(30.0)	28(30.4)	5(27.8)		
			Unemployed	5(4.5)	3(3.3)	2(11.1)		
			Artisan	36(32.7)	30(32.6)	6(33.3)		
			Retired	14(12.7)	12(13.0)	2(11.1)		
			Civil servant	20(18.2)	17(18.4)	3(16.7)		
Level of Education							14.052	0.003
			None	10(9.1)	5(5.4)	5(27.8)		
			Primary	25(22.7)	19(20.6)	6(33.3)		
			Secondary	40(36.4)	34(36.9)	6(33.3)		
			Tertiary	35(31.8)	34(37.0)	1(5.6)		

Symptomatology observed in the patients

The most common symptoms in the patients were malaise, leg swelling, jaundice, and weight loss, which were seen in 78 (70.9%), 69 (62.7%), 64 (58.2%), and 57 (51.8%) patients respectively. However, malaise and abdominal swelling were significantly more frequent in patients who died compared to those who survived (p= 0.016 and p= 0.007) respectively, whereas the reverse was the case for leg swelling (p= 0.079) (Table 2).

**Table 2:** Symptomatology observed in patients.

Symptoms			Total patients	Outcome of liver cirrhosis		ꭓ^2^	p-value
			(n=110)	Survived (n=92)	Died (n=18)		
Jaundice						3.397	0.065
		Yes	64(58.2)	50(54.3)	14(77.8)		
		No	46(41.8)	42(45.7)	4(22.2)		
Malaise						5.779	0.016
		Yes	78(70.9)	61(66.3)	17(94.4)		
		No	32(29.1)	31(33.7)	1(5.6)		
Leg swelling						3.077	0.079
		Yes	69(62.7)	61(66.3)	8(44.4)		
		No	41(37.3)	31(33.7)	10(55.6)		
Weight loss						3.589	0.058
		Yes	57(51.8)	44(47.8)	13(72.2)		
		No	53(48.2)	48(52.2)	5(27.8)		
Abdominal swelling						7.905	0.007
	Yes		65(59.1)	49(53.2)	16(88.9)		
	No		45(40.1)	43(46.7)	2(11.1)		
Haemetemesis						19.957	0.001
		Yes	11(10.0)	4(4.3)	7(38.9)		
		No	99(90.0)	88(95.7)	11(61.1)		
Meleana						14.361	0.016
		Yes	78(70.9)	61(66.3)	17(94.4)		
		No	32(29.1)	31(33.7)	1(5.6)		

Signs observed in the patients

Table 3 shows the signs seen in the patients recruited. The most frequent signs were ascites, pedal edema, abdominal distension, and jaundice which were seen in 75 (68.1%),69(62.7%), 66(60%), and 63(57.3%) patients respectively. However, moderate ascites, splenomegaly, and grade 4 hepatic-encephalopathy were significantly more present in patients who died compared to those that survived (p = 0.001, 0.001, and 0.012 respectively), but the reverse was the case for abdominal distension (p = 0.027).

**Table 3:** Signs observed in patients.

Signs		Total patients	Outcome of liver cirrhosis		ꭓ^2^	p-value
		(n=110)	Survived (n=92)	Died (n=18)		
PalIor					8.730	0.003
Yes		45(40.9)	32(34.8)	13(72.2)		
No		65(59.0)	60(65.2)	5(27.8)		
Pedal oedema					0.143	0.794
Yes		69(62.7)	57(62.0)	12(66.7)		
No		41(37.3)	35(38.0)	6(33.3)		
Jaundice					3.698	0.054
Yes		63(57.3)	49(53.3)	14(77.8)		
No		47(42.7)	43(46.7)	4(22.2)		
Parotid fullness					1.684	0.302
Yes		2(1.8)	1(5.6)	1(5.6)		
No		108(98.2)	91(98.9)	17(94.4)		
Wasting of small muscles of the hand					1.274	0.259
Yes		42(38.2)	33(35.9)	9(50.0)		
No		68(61.8)	59(64.1)	9(50.0)		
Clubbing					0.082	0.775
Yes		21(19.1)	18(19.6)	3(16.7)		
No		89(80.9)	74(80.4)	15(83.3)		
Ascites					43.619	0.001
Mild		33(30.0)	32(37.7)	1(5.6)		
Moderate		35(31.8)	25(27.2)	10(55.6)		
Severe		7(6.4)	1 (1.1)	6(33.3)		
No ascites		34(30.9)	33(37.0)	1(5.6)		
Hepatomegaly					1.747	0.240
Yes		14(12.7)	10(10.9)	4(22.2)		
No		96(87.3)	82(89.1)	14(77.8)		
Splenomegaly					39.792	0.001
Yes		18(16.4)	6(6.5)	12(66.7)		
No		92(83.6)	86(93.5)	6(33.3)		
Distended abdominal veins (%)					0.001	0.976
Yes		12(0.9)	10(10.9)	2(11.1)		
No		98(89.1)	82(89.1)	16(88.9)		
Hepatic-encephalopathy					6.663	0.012
	Absent	3(2.7)	2(2.1)	1(5.6)		
	Grade 0/Minimal	28(25.5)	28(30.4)	0(0.0)		
	Grade 1	28(25.5)	26(28.3)	2(11.1)		
	Grade 2	21(19.1)	19(20.7)	2(11.1)		
	Grade 3	17(15.4)	13(14.1)	4(22.2)		
	Grade 4	13(11.8)	4(4.3)	9(50.0)		
Abdominal distension					4.88	0.027
	YES	66(60)	51(55.4)	15(83.3)		
	NO	44(40)	41(44.6)	3(16.7)		

Features of decompensation observed in patients are outlined in Table 4, most of the patients had encephalopathy followed by ascites and jaundice.

**Table 4:** Features of decompensation in patients. Multiple responses

Features	Number of patients
Jaundice	63
Ascites	75
Encephalopathy	107
Coagulopathy	48
Splenomegaly	18

 Risk factors for liver cirrhosis

The most frequent risk factor was alcohol, which was found in 69 (62.7%) patients. This was followed by HBV infection in 28 (25.5%). However, only alcohol and family history of liver disease were significantly associated with mortality (p < 0.05). Significant alcohol intake was considered as greater than 21 units for men and greater than 14 units for women (Table 5).

**Table 5:** Risk factors for liver cirrhosis. HBV, hepatitis B virus; HCV, hepatitis C virus

Risk factors	Total patients (n=110)	Outcome of liver cirrhosis		ꭓ^2^	p-value
		Survived (n=92)	Died (n=18)		
Alcohol				3.909	0.048
Yes	69(62.7)	54(58.7)	15(83.3)		
No	41(37.3)	38(41.3)	3(16.7)		
HBV infection				0.876	0.349
Yes	28(25.5)	25(27.2)	3(16.7)		
No	82(74.5)	67(72.8)	15(83.3)		
HCV infection				0.540	0.463
Yes	18(16.4)	14(15.2)	4(22.2)		
No	92(83.6)	78(84.8)	14(77.8)		
Family history of liver disease (%)				4.389	0.036
Yes	14(12.7)	9(9.8)	5(27.8)		
No	96(87.3)	83(90.2)	13(72.2)		

Comparison of baseline laboratory parameters and scores between the survivors and the patients who died at 1 month

Table 6 compares the mean values of laboratory parameters at presentation. The mean packed cell volume (PCV), platelet count, albumin, and serum sodium were significantly lower (p < 0.05), while the mean values of AST, ALT, PT, and creatinine were significantly higher (p < 0.05) among non-survivors compared to the survivors at 1 month.

**Table 6:** Comparison of baseline laboratory parameters and scores between the survivors and the patients who died at 1 month. PCV, packed cell volume; PT, prothrombim time; INR, international normalized ratio; ALT, alanine transaminase; AST, aspartate transaminase

Laboratory parameters	Liver disease		t-test	p-value
	Survived (n=92) (mean)	Died (n=18) (Mean)		
Hematological				
PCV, %	28.12 ± 3.18	27.0 ± 1.64	1.502	0.028
Platelet count, mm^3^	204706 ± 72094	133833 ± 35402	4.065	0.001
PT, s	20.49 ± 5.39	24.277 ± 6.83	0.092	0.020
INR	2.77 ± 0.74	2.27 ± 0.64	2.695	0.008
Liver function tests				
ALT, IU/L	61.37 ± 58.38	91.94 ± 63.33	2.002	0.048
AST, IU/L	84.78 ± 78.68	124.78 ± 70.87	2.002	0.048
Total bilirubin	3.65 ± 5.23	4.50 ± 7.02	0.592	0.555
Albumin	2.14 ± 0.67	1.5 ± 0.27	0.788	0.032
Serum sodium	134.17 ± 4.92	131.22 ± 3.92	2.375	0.019
Serum creatinine	1.032 ± 0.470	2.99 ± 0.951	13.258	0.001

MELD score and mortality

Higher mortality (88.9%) was observed among patients with a MELD score of ≥ 20, compared to those with a MELD score of <20 (11.1%) (Table 7).

**Table 7:** MELD scoring of the study patients.

MELD score	Outcome of liver cirrhosis			ꭓ^2^	p-value
	Total	Survived (n=92)	Dead (n=18)		
<20	57(51.8)	55(59.8)	2(11.1)	14.244	0.001
≥20	53(48.2)	37(40.2)	16(88.9)		

Predictors of 1-month mortality of decompensated liver cirrhosis

**Figure 1:**
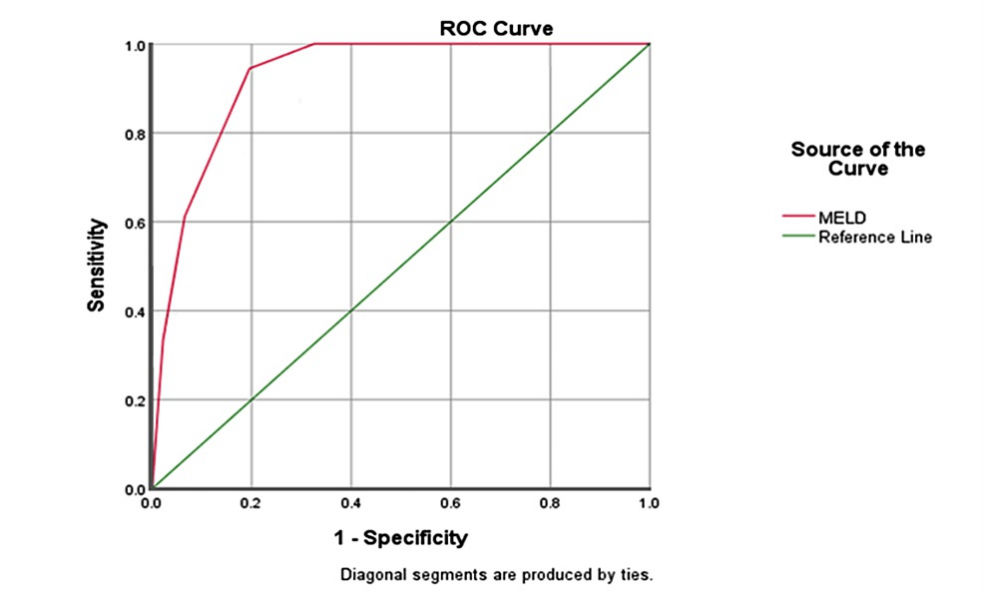
ROC curves for the predictive values of MELD score for 1-month mortality. ROC, receiver operating characteristic

A multivariate logistic regression done to determine the predictors of mortality of liver disease over one month showed that older age, degree of encephalopathy, degree of ascites, low serum sodium, elevated serum creatinine, prolonged INR, and low serum albumin were predictors of mortality (p < 0.05) as shown in Table 8. Also, MELD score was found to be an independent predictor of mortality at the end of the follow-up score (odds ratio, OR=1.28, confidence interval, CI: 1.16-1.41) was noticed to predict mortality with high sensitivity (72.2%) and PPV (93.6%) as shown in Figure 1.

**Table 8:** Multiple logistic regression analysis showing various clinical, laboratory variables, and scoring systems at admission as independent predictors of 1-month mortality in the patients. MELD, Model for End-Stage Liver Disease; INR, international normalized ratio; PT, prothrombin time; OR, odds ratio; CI, confidence interval; PCV, packed cell volume *Chi-square

Predictors of liver cirrhosis 1-month mortality	Outcome of liver cirrhosis		OR (95% CI)	t-test	p-value
	Survived (n=92)	Died (n=18)			
Age (yrs)	51.52 ± 12.39	62.67 ± 9.73	1.09(1.01-1.17)	3.600	0.001
Encephalopathy (%)			1.25(1.01-1.20)	6.663*	0.012
Grade 0/minimal	2(2.1)	1(5.6)			
Grade 1	26(32.6)	2(11.1)			
Grade 2	19(28.3)	2(11.1)			
Grade 3	13(20.7)	4(22.2)			
Grade 4	4(4.3)	9(50.0)			
NO	30(32.6)	1(5.6)			
Ascites (%)			1.50(1.01-1.45)	43.619*	0.001
Mild	32(37.7)	1(5.6)			
Moderate	25(27.2)	10(55.6)			
Severe	1(1.1)	6(33.3)			
No ascites	34(37.0)	1(5.6)			
PCV (%)	28.12 ± 3.18	26.0 ± 1.64	0.45(0.15-1.39)	1.502	0.028
Sodium, mmol/L	132.17 ± 4.92	131.22±3.92	1.10(0.79-0.98)	2.375	0.019
Serum creatinine, mg/dL	1.032 ± 0.470	2.99 ± 0.951	1.20(0.87-1.05)	13.258	0.001
INR	2.26 ± 0.74	3.07 ± 0.64	1.40(1,15-2.5)	2.695	0.008
Albumin, g/L	2.14 ± 0.67	1.5 ± 0.27	2.56(0.24-2.8)	0.788	0.032
PT, s	20.49 ± 5.39	24.277 ± 6.83	0.83(0.61-1.14)	0.092	0.020
MELD score	18.52 ± 5.38	26.76 ± 5.20	1.28(1.16-1.41)	0.045	0.001

## Discussion

A total of 110 patients with decompensated liver cirrhosis were recruited for this study; they were analyzed and grouped into survivors and those that died within a 1-month follow-up period. A mortality rate of 16.4% was obtained.

In this study, alcohol intake was noted to be the most common risk factor (62.7%) for liver cirrhosis. Most of the patients who died during the course of the study had significant alcohol intake (83.3%). Nwokediuko et al. [10] reported similar results where alcohol intake (52.7%) was the most common risk factor for liver cirrhosis in their study. An association between increased alcohol intake and mortality was reported by Udompap et al. [7]. HBV and HCV infections were reported by Attia et al. [24] and Fayad et al. [20] as the most common risk factors respectively. This difference in risk factors may be due to the increased intake of alcohol in the South-South region of Nigeria.

The mean age of the patients with decompensated liver cirrhosis was 57 ± 11.06 years with a male preponderance (M:F of 2.9:1). Similar findings were reported in a study conducted by Aldawood and colleagues [25] in Saudi Arabia where the mean age of the patients in that study was 59.8 ± 14.1 years with a male preponderance (M: F of 2.1). However, contrasting results were reported from the studies done by Apica et al. [12] in Uganda and Peng et al. [19] in China who reported lower mean ages of 45 ± 12.5 and 50.5 ± 7.02 years respectively.

Patients who died during the course of this study had a mean age of 62.7 ± 9.7 years. This is slightly lower than 64 ± 12.5 years reported by Suman et al. [26] in their study done in India. Deterioration of liver function associated with aging may account for the relationship between mortality and the older age group. Most of the patients who died were artisans and those with no formal education. This may be explained by the fact that these groups of patients belong to the low socioeconomic class and have poor health behavior, and may also have compromised nutritional status.

Malaise (94.4%) and hematemesis (38.9%) were the most common features among those that died. Also, most patients who died had moderate ascites (61.9%) and grade 4 encephalopathy (50.0%). This is comparable to the study done in India by Acharya et al. [27] who found ascites, hematemesis, hepatic encephalopathy, and peripheral edema to occur significantly more among non-survivors than those who survived. This may be explained by an increase in susceptibility to bacterial infections leading to deterioration in hepatic function.

Using the multivariate logistic regression, prolonged PT, INR, high MELD score, older age, severe ascites, elevated serum bilirubin, and low serum sodium was found to be predictive of mortality in patients with decompensated liver cirrhosis. Hepatic encephalopathy was also found to be an independent predictor of mortality in this study. This has also been demonstrated in other studies by Okonkwo et al. [28] in Nigeria and Acharya et al. [27] in India who observed that there is a significant association between hepatic encephalopathy and mortality.

The MELD scores have been widely used for the assessment of prognosis in liver cirrhosis. This study shows that MELD scores are valid predictors of mortality from decompensated liver cirrhosis irrespective of the etiology. The MELD score was demonstrated in the course of this study to have high predictive ability for mortality in decompensated liver cirrhosis. Okwonkwo et al. [28] and Papatheodoridis et al. [29] also demonstrated that MELD score could accurately predict short-term outcome in patients with decompensated liver cirrhosis.

With a sensitivity of 72.2% and PPV of 93.6% and area under curve (AUC) of 0.926 in this study, MELD score was found to be highly predictive of mortality in patients with decompensated liver disease. This is similar to the findings by Okonkwo et al. [28] who also reported a high sensitivity (80%) and AUC values for MELD score.

Overall, the MELD score has been found to be well suited for prioritizing patients who would benefit from liver transplantation.

Limitation of study

Some of the patients in this study who required liver biopsy to confirm their diagnosis could not get it done due to the presence of contraindications in them and refusal by some.

## Conclusions

The MELD score was found to have high sensitivity, specificity, and PPV to predict 1-month mortality among the patients with decompensated liver cirrhosis. The MELD score should be used readily in assessing severity and mortality in patients with liver cirrhosis. Patients with liver cirrhosis with MELD score ≥20 should be regarded as high risk for mortality.
